# PAFAH1B3 predicts poor prognosis and promotes progression in lung adenocarcinoma

**DOI:** 10.1186/s12885-022-09617-x

**Published:** 2022-05-09

**Authors:** Suping Tang, Jun Ni, Bohua Chen, Fei Sun, Jinbo Huang, Songshi Ni, Zhiyuan Tang

**Affiliations:** 1 Department of Respiratory and Critical Care Medicine, Affiliated Hospital of Nantong University, Nantong, 226001 Jiangsu China; 2 Department of Respiratory and Critical Care Medicine, The Affiliated Wuxi Second People’s Hospital of Nanjing Medical University, Wuxi, 214000 Jiangsu China; 3 Department of Rehabilitation Medicine, The First Affiliated Hospital of Fujian Medical University, Fuzhou, 350000 Fujian China; 4 Department of Rehabilitation Medicine, Affiliated Hospital of Nantong University, Nantong, 226001 Jiangsu China; 5 Department of Pharmacy, Affiliated Hospital of Nantong University, Nantong, 226001 Jiangsu China

**Keywords:** PAFAH1B3, LUAD, Prognosis, EMT, Immune infiltrates

## Abstract

**Background:**

Recently, increasing evidence has indicated that platelet-activating factor acetylhydrolase 1b catalytic subunit 3 (PAFAH1B3) plays an important role in several cancers. However, its role in lung adenocarcinoma (LUAD) has not been reported until now.

**Methods:**

The expression of PAFAH1B3 in LUAD was determined by using the Gene Expression Profiling Interactive Analysis (GEPIA) database and real-time PCR (RT–PCR), western blot and immunohistochemical (IHC) analyses. A chi-square test was used to investigate the correlation between PAFAH1B3 expression and clinical parameters. Cox regression and Kaplan–Meier analysis were performed to analyze the prognostic value of PAFAH1B3. The CCK-8 assay, clone formation assay, transwell invasion assay and flow cytometry were conducted to detect cell proliferation, clone formation, invasion and the cell cycle. The xenograft tumor model was constructed to explore the function of PAFAH1B3 *in vivo*. Western blot and IHC analyses were performed to detect epithelial-to-mesenchymal transition (EMT)-related markers. Immune Cell Abundance Identifier (ImmuneCellAI) and IHC analyses were used to analyze the effect of PAFAH1B3 on immune cell infiltration.

**Results:**

Our study showed that the expression of PAFAH1B3 was upregulated in LUAD tissues and cells compared with noncancerous tissues and cells. Additionally, the results indicated that the expression of PAFAH1B3 was positively correlated with distant metastasis, TNM stage and poor clinical outcome and it was an independent prognostic risk factor for LUAD. In addition, silencing PAFAH1B3 suppressed cell proliferation, colony formation, and invasion and increased the cell population in the G0-G1 phases *in vitro*. Furthermore, our results showed that knockdown of PAFAH1B3 increased the epithelial marker E-cadherin level and decreased the mesenchymal marker N-cadherin level *in vitro* and *in vivo*. We also proved that PAFAH1B3 downregulation inhibited tumorigenesis and neutrophil infiltration in the xenograft tumor model.

**Conclusion:**

Our studies indicate that PAFAH1B3, a prognostic risk factor, promotes proliferation, invasion and EMT and affects immune infiltrates in LUAD.

**Supplementary Information:**

The online version contains supplementary material available at 10.1186/s12885-022-09617-x.

## Background

Lung cancer has become the most commonly diagnosed malignancy and is the leading cause of cancer-related mortality among all cancers [1]. Non-small-cell lung cancer (NSCLC) is the most common pathological type of lung cancer, accounting for 85% of all lung cancer cases. There are three main subtypes of NSCLC: adenocarcinoma, squamous-cell carcinoma, and large-cell carcinoma, accounting for 40%, 25–30%, and 10–15% of all lung cancer cases, respectively [2]. Although significant progress has been made in the early diagnosis, targeted therapy and immunotherapy of NSCLC in recent years, the reality is that the overall survival (OS) of lung adenocarcinoma (LUAD) patients is still low [3, 4]. Therefore, accurate diagnostic, prognostic markers and effective therapeutic targets and predictors of response to immunotherapy for LUAD are urgently needed.

Platelet-activating factor acetylhydrolases (PAF-AHs) are a group of structurally variable isoenzymes that can catalyze the hydrolysis of the sn-2 acetyl group of platelet-activating factor (PAF), which is a lipid mediator participating in various physical and pathological processes, such as apoptosis, angiogenesis, the inflammatory response, wound healing and tumor development [5–7]. PAF-AHs are divided into three types, plasma PAF-AH and intracellular PAF-AHI and PAF-AHII, among which PAF-AHI has been recently recognized as an oncogenic factor [5].

Platelet-activating factor acetylhydrolase 1b catalytic subunit 3 (PAFAH1B3), is a protein-coding gene that encodes the 29 kDa catalytic subunit, namely, the α1 subunit of PAF-AHI [8]. Many studies have found that PAFAH1B3 plays an important role in various cancers. PAFAH1B3 is considered an oncogene and is upregulated in several cancers, including prostate cancer, melanoma, breast cancer and ovarian cancer [9]. P11, an inhibitor of PAFAH1B2 and PAFAH1B3, can impair the pathogenicity of these cancers [9, 10]. In addition, through metabolic profiling, PAFAH1B3 expression has been found to be upregulated in breast carcinoma cells, and downregulation of PAFAH1B3 remarkably inhibits breast carcinoma cell proliferation, migration and invasion [11]. Moreover, PAFAH1B3 loss sensitizes acute lymphoblastic leukemia cells to dasatinib *in vivo* [12]. Furthermore, PAFAH1B3 is associated with clinical outcome and contributes to the progression of hypopharyngeal squamous cell carcinoma (HSCC) [13]. However, no studies have confirmed the precise function of PAFAH1B3 in LUAD.

Tumor-infiltrating immune cells have been found to correlate with the survival of patients with different solid tumors. For example, a previous study showed that tumor-infiltrating CD8+ T lymphocytes are a favorable factor in patients with breast cancer [14]. Similarly, higher numbers of tumor-associated macrophages have been proven to exacerbate tumor progression and be associated with worse clinical prognosis in multiple cancers [15]. Furthermore, the function of tumor-infiltrating B lymphocytes remains controversial, as some studies have shown that B cells have antitumor capacity, while others indicate that different immunosuppressive subtypes of B cells may exert protumoral effects [16]. However, the exact mechanism of immune infiltration in LUAD remains unknown.

Thus, in this study, we aimed to demonstrate the correlations between the PAFAH1B3 expression level and clinicopathological parameters and to confirm the potential of PAFAH1B3 as a prognostic biomarker in LUAD patients. We also attempted to examine the function of PAFAH1B3 in proliferation and invasion *in vitro* and *in vivo*. Furthermore, we attempted to explain the relationship between PAFAH1B3 expression levels and epithelial-to-mesenchymal transition (EMT)-related protein expression levels. Finally, we attempted to find an association between PAFAH1B3 and tumor immune infiltration.

## Materials and methods

### Patients and tumor specimens

We collected 8 primary LUAD tissues and paired adjacent noncancerous tissues from patients undergoing surgical treatment with their written informed consent. The surgically removed tissues were immediately frozen in liquid nitrogen and stored at −80 °C. The lung adenocarcinoma tissue microarray was purchased from Superbioteck (Shanghai, China) which contained 79 tumor tissues and 77 paired adjacent noncancerous tissues. All samples had been pathologically diagnosed as lung adenocarcinoma. This experiment was approved by the Ethics Committee of Affiliated Hospital of Nantong University (Ethic Number: 2018-K020).

### TCGA data acquisition

The LUAD RNA-seq and corresponding clinical data were downloaded from the Cancer Genome Atlas (TCGA, http://cancergenome.nih.gov/) database, which including 535 tumor samples and 59 paired adjacent noncancerous samples. Cases with missing information on age, survival information, T classification, N classification and TNM stage were excluded and 475 tumor cases were included in Cox regression analysis. We kept 535 tumor samples for ImmuCellAI to explore the influence of PAFAH1B3 on the immune microenvironment of LUAD.

### GEPIA

Gene Expression Profiling Interactive Analysis (GEPIA, http://gepia.cancer-pku.cn/) database was used to analyze the differential expression of PAFAH1B3 in 483 LUAD samples and 347 normal samples from the TCGA and the GTEx projects. We also conducted disease free survival (DFS) and overall survival (OS) analysis by using the “Survival” module of GEPIA.

### Immunohistochemical (IHC) analyses

After dewaxing and rehydrating, paraffin sections were treated with 3% hydrogen peroxide to eradicate endogenous peroxidase. The tissue sections were placed in the antigen repair solution and were boiled in a microwave oven for antigen repair retrieval. Then, 5–10% goat serum was used to blocking nonspecific binding site and tissue sections were incubated with the following antibody: Anti-PAFAH1B3 (1:500, Invitrogen, MA5–26672), Anti-E-cadherin (1:1000, Servicebio, GB12083), Anti-N-cadherin (1:1000, Servicebio, GB111273), Anti-SNAIL (1:500, Servicebio, GB11260), Anti-Ly6g (1:500, Servicebio, GB11229) overnight at 4 °C. After, incubating with secondary antibody, diaminobenzidine (DAB) solution was used for immunohistochemical staining. Two independent-experienced pathologists evaluated staining intensity and percentage of positive cells of all tissue sections double blindly. The straining intensity was categorized into 4 levels: 0 (negative), 1 (weak), 2 (medium), and 3 (strong), percentage of positive tumor cells was categorized into 4 levels: 1 ((≤25%)), 2 (26–50%), 3 (51–75%), and 4 (>75%). Immunoreactive score (IRS) = staining intensity × percentage of positive cells. Then the X-file software (Rimm Laboratory at Yale University, http://www.tissuearray.org/rimmlab) was used to divide the PAFAH1B3 protein expression into two categories (high expression and low expression). Here, IRS value>6 was considered as a high expression.

### Cell culture

Human bronchial epithelial cell line (16HBE) and Human LUAD cell lines (NCI-H1299, NCI-H1650, A549, SPCA1) were all from Cell Bank of Chinese Academy of Science (Shanghai, China). 16HBE and LUAD cells were grown in DMEM medium (Biological Industries, Israel) and RPMI-1640 medium (Biological Industries, Israel), respectively. All medium were supplemented with 10% FBS (Biological Industries, Israel), 100 U/mL penicillin (Beyotime, china) and 0.1 mg/mL streptomycin (Beyotime, china). All cells were cultured at 37 °C in a 5% CO2 humidified atmosphere.

### RNA isolation and real-time PCR (RT-PCR)

Total RNA was isolated from cells with Trizol reagent (Invitrogen, USA) and reverse transcribed to cDNA using RevertAid First Strand cDNA Synthesis Kit (Thermo Fisher, USA). cDNA was then amplified using FastStart Universal SYBR Green Master (ROX) (Roche, Switzerland) by ABI 7500 (Applied Biosystems, USA). GAPDH was used for normalization. The gene expression was quantified using the 2^-ΔΔ Ct^ method. The primer sequences were as follows: PAFAH1B3 F: 5′-CTGGGCTACACACCTGTTTGC-3′, PAFAH1B3 R 5′-GGAGAGTTTAATGTTGTGGGAAGG-3′, GAPDH F: 5′-GAACGGGAAGCTCACTGG-3′, GAPDH R: 5′-GCCTGCTTCACCACCTTCT-3′.

### Western blot

To extract proteins, we treated cells or tissues with RIPA Lysis Buffer (Beyotime, China) which containing phenylmethanesulfonyl fluoride (PMSF) (Beyotime, China). The protein concentration was measured by the BCA kit (Beyotime, China) and 20ug of total protein was added to each well for protein separation by SDS-PAGE. Then the protein was transfered to PVDF membranes (Millipore, USA), blocked with 5% nonfat milk and incubated with primary and secondary antibodies for immunoblotting. Finally, the protein bands were detected by the ECL chemiluminescent substrate kit (Biosharp, china). The primary antibodies were as follows: PAFAH1B3 rabbit monoclonal (1:1000, ab166906, Abcam), E-cadherin rabbit monoclonal (1:10000, ab40772, Abcam), N-cadherin rabbit monoclonal (1:5000, ab76011, Abcam), SNAIL+SLUG rabbit monoclonal (1:1000, ab85936, Abcam). GAPDH mouse monoclonal (1:100000, 60,004–1-Ig, Proteintech). The secondary antibodies were: anti-rabbit secondary antibody (HRP) (1:5000, AB0101, Abways Technology), anti-mouse secondary antibody (HRP) (1:2000, AB0102, Abways Technology).

### Gene knockdown and overexpression by lentiviral transfection

Lentivirus-mediated short hairpin RNA (shRNA) targeting PAFAH1B3 and lentivirus overexpressing PAFAH1B3 were acquired from Shanghai Gene Pharma Co, Ltd., China. A549, H1299, and SPCA1 cells were seeded in 6-well plates at a density of 8 × 10^4^ cells per well overnight. A549 and H1299 cells were transfected with lentivirus to produce stable PAFAH1B3-silenced cell lines and SPCA1 cells were transfected with lentivirus to produce stable PAFAH1B3-enhanced cell lines. After 72 h, cells were cultured with medium containing puromycin (Beyotime, china**)** to kill cells that had not been transfected with lentivirus. The efficiency of silencing PAFAH1B3 was detected by western blot and the efficiency of overexpression PAFAH1B3 was detected by RT–PCR. A scrambled shRNA (shControl) was used as a control. The sequences of three lentivirus-mediated shRNAs targeting PAFAH1B3 were as follows: (shRNA1: 5′-gaTGGCACCATCAGCCATCAT-3′, shRNA2: 5′-cgACAGGTGAACGAGCTGGTA-3′, shRNA3: 5′-gcAGGTGACTGGTGGCATCAA-3′) and the sequences of control shRNA was 5′-TTCTCCGAACGTGTCACGT-3′.

### Cell viability assay

Cells were seeded in the 96-well plates at a density of 2 × 10^3^ per well. At 24 h, 48 h, 72 h, 96 h, the medium was removed and replaced with 110ul medium containing 10ul counting kit-8 (CCK-8) solution. After continuing to incubate for 2 hours, the absorbance at 450 nm were measured.

### Colony formation assay

Cells were seeded in 6-well plates at a density of 1 × 10^3^ per well and the medium was changed every 3 days. After 9 days, the original medium was removed, the cells were washed twice with PBS and fixed with 4% paraformaldehyde for 20 minutes. Then cells were stained with crystal violet for 5 minutes. Finally, the cells were washed for 3 times with PBS and pictured.

### Cell invasion assay

The transwell chambers (8um, Corning, USA) were coated with 50ul of 1 mg/mL Matrigel (356,234, BD Sciences, USA). We seeded 5 × 10^4^ cells in the upper chamber in serum-free medium while medium containing 20% FBS was added to the lower chamber. After 48 hours of incubation, non-invaded cells were swabbed off and invaded cells were fixed with 4% paraformaldehyde for 20 minutes and stained with 0.5% crystal violet for 5 minutes. Finally, cells were photographed (200 × magnification) at five random views and counted with ImageJ.

### Cell cycle analysis

Cells in 6-well plate at logarithmic growth stage were collected, washed with pre-cooled PBS and then fixed in pre-cooled 75% alcohol for 2 hours. Then cells were were stained with propidium iodide (PI) staining solution (US Everbright® Inc., china) containing RNase A. After incubating for 30 minutes at room temperature and protecting from light, the cell populations were detected by flow cytometry at 615 nm emission wavelength (BD Biosciences, USA) and analyzed with ModFit LT.

### ImmuCellAI

Immune Cell Abundance Identifier (ImmuCellAI) (http://bioinfo.life.hust.edu.cn/web/ImmuCellAI/) is a web server which was applied to estimate the abundance of 24 immune infiltrating cells [17]. Compared to other methods, ImmuCellAI can accurately evaluate the abundance of immune cells especially on multiple T-cell subpopulations. According to PAFAH1B3 expression of the 535 LUAD samples from TCGA database, the upper 1/3 samples were included into PAFAH1B3-high expression group while the lower 1/3 were included into the PAFAH1B3-low expression group. Then we upload the file to ImmuCellAI to analysis the immune infiltration level.

### Mouse xenograft tumor experiment

Male nude mice (6-week-old) were purchased from the Shanghai Experimental Animal Center, Chinese Academy of Sciences, Shanghai, China. We injected 5 × 106 cells in 100 ul PBS subcutaneously into the right flanks of the mice. After tumor formation, tumor size was measured every three days and tumor volume was calculated according to the formula V (mm^3^) = with^2^ × length×0.52. After 28 days later, the tumors were harvested, measured for size and photographed. Part of the tumor tissues were placed in 10% formalin for immunohistochemistry and examined by pathology staff. The remaining tumors were frozen in −80 °C for subsequent experiments. All the experiments were performed according to the guidelines for the care and use of laboratory animals. All the animal experimental protocols were in accordance with ARRIVE guidelines and approved by the Ethics Committee of the Affiliated Hospital of Nantong University (Ethic Number: S20210225–032).

### Statistical analysis

SPSS 16.0 software (IBM) and Graphpad Prism 7.0 (GraphPad Software) were used for all the statistical analysis. The data were shown as mean ± (standard deviation) SD from at least three independent experiments. Paired t-test was used for paired tissue samples. Student’s t-test was used for two groups. The chi-square test was used to analysis the relations between PAFAH1B3 and clinical features. Cox regression was used for univariate and multivariate analysis of prognostic factors. *P* < 0.05 was considered significant.

## Results

### PAFAH1B3 expression is upregulated in LUAD tissues and cell lines

To determine PAFAH1B3 expression in LUAD, we analyzed PAFAH1B3 mRNA expression data from the Gene Expression Profiling Interactive Analysis (GEPIA) database. We found that PAFAH1B3 mRNA was remarkably overexpressed in LUAD tissues (483 samples) compared with noncancerous lung tissues (347 samples) (Additional file 1: Fig. S1a, *P* < 0.05). To exclude individual differences, we further analyzed the mRNA expression of PAFAH1B3 in 57 paired lung tumor and adjacent noncancerous tissues in TCGA LUAD datasets. We found that PAFAH1B3 expression was significantly upregulated in 52 lung tumor samples compared with matched adjacent noncancerous samples (Additional file 1: Fig. S1b, *P* < 0.01).

**Fig. 1:**
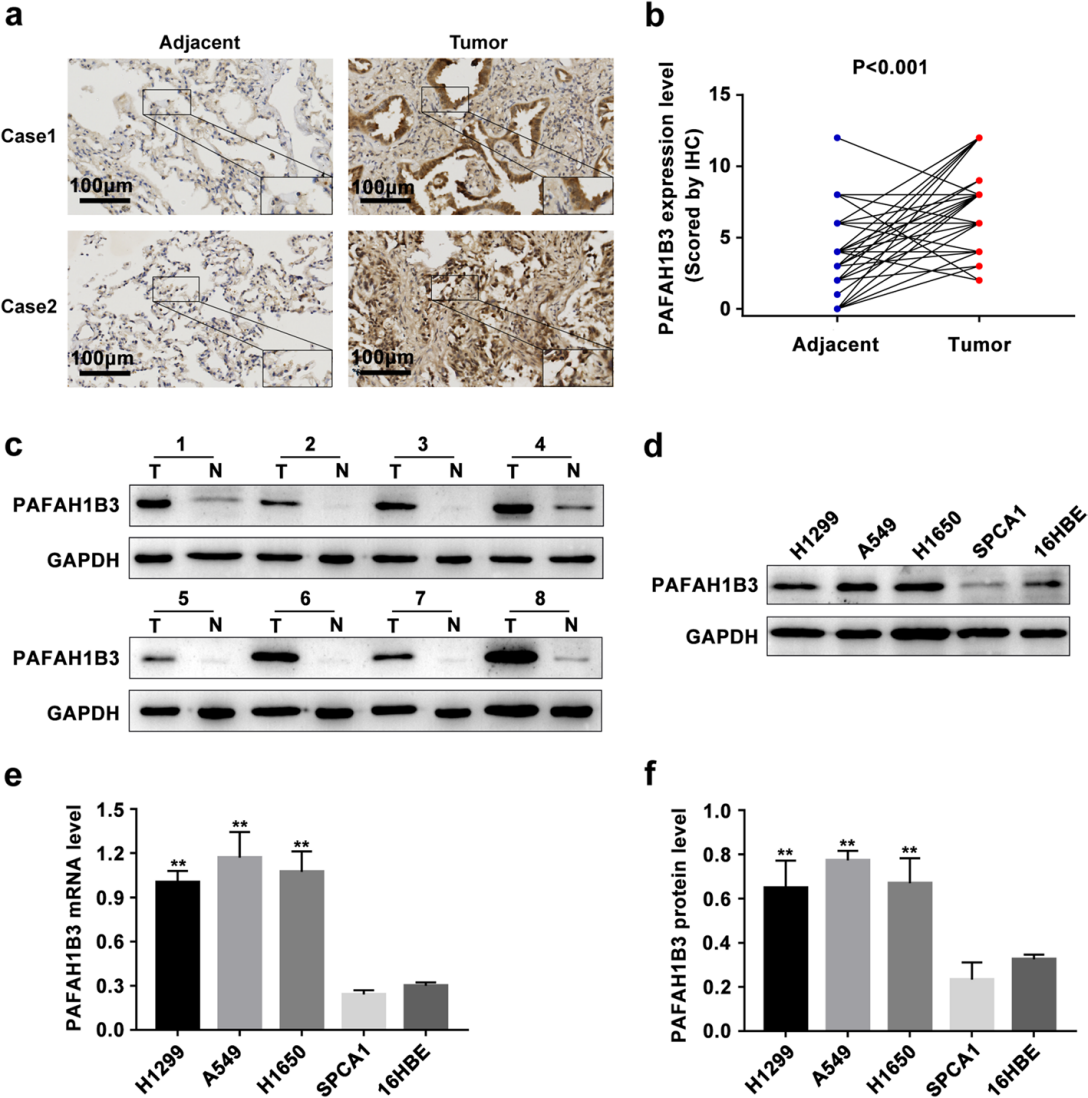
PAFAH1B3 is up-regulated in LUAD tissues and cells. **a** Representative immunohistochemical staining pictures for PAFAH1B3 in LUAD tissues and adjacent noncanerous tissues. **b** IHC analyses score of PAFAH1B3 protein expression in 77 paired tumor tissues and adjacent noncancerous tissues in LUAD tissue microarray. **c** PAFAH1B3 protein expression in eight paired LUAD tissues (T) and their adjacent noncancerous tissues (N) by western blot. **d, f** PAFAH1B3 protein expression in 16HBE and four LUAD cell lines by western blot. **e** PAFAH1B3 mRNA expression in 16HBE and four LUAD cell lines by RT–PCR. * *P* < 0.05, ** *P* < 0.01

To further determine whether PAFAH1B3 expression is indeed increased in LUAD tissues, we first analyzed PAFAH1B3 protein levels in four human LUAD cell lines (H1299, A549, H1650 and SPCA1) and found that both the protein and mRNA expression levels of PAFAH1B3 were remarkably upregulated in A549, H1299 and H1650 cells compared with 16HBE cells (Fig. 1d, e, f, *P* < 0.01). Next, we quantified the PAFAH1B3 protein levels of 77 pairs of LUAD samples and adjacent nontumor samples in the tissue microarray by immunohistochemistry (IHC) analyses. Our data showed that PAFAH1B3 was mainly localized in the cytoplasm of cancer cells and that the PAFAH1B3 protein level was prominently higher in 65 LUAD tissues than in paired adjacent noncanerous tissues (Fig. 1a, b, *P* < 0.01). We also collected eight pairs of paired primary LUAD tissues and adjacent noncancerous tissues. Western blot revealed that the PAFAH1B3 protein level was significantly upregulated in the human primary LUAD tissues compared with paired adjacent noncancerous tissues (Fig. 1c, *P* < 0.01). The above results indicate that PAFAH1B3 is upregulated and may be an oncogene in human LUAD.

### PAFAH1B3 has prognostic value in LUAD patients

To investigate whether PAFAH1B3 expression is associated with the clinical outcome of LUAD patients, we generated survival curves by using the “Survival” module of GEPIA database. The results suggested that higher PAFAH1B3 expression correlated with shorter OS and disease-free survival (DFS) durations in LUAD patients (Additional file 1: Fig. S2a, b, *P* < 0.01). Furthermore, according to the gene expression level of PAFAH1B3, we classified the 475 primary LUAD samples from TCGA database into two categories by the median cutoff: PAFAH1B3 low (n = 237) and PAFAH1B3 high (n = 238). Univariate analysis showed that the expression of PAFAH1B3 (HR = 1.497, *P* = 0.009, 95% CI = 1.018–2.023), primary tumor (HR = 1.690, *P* < 0.001, 95% CI = 1.336–2.139), lymph node metastasis (HR = 2.496, *P* < 0.001, 95% CI = 1.851–3.366) and TNM stage (HR = 1.804, *P* < 0.001, 95% CI = 1.517–2.145) were significantly associated with the risk of death (Additional file 1: Table S1). In the multivariate analysis, PAFAH1B3 expression level (HR = 1.416, *P* = 0.024, 95% CI = 1.047–1.915) and TNM stage (HR = 1.782, *P* < 0.001, 95% CI = 1.498–2.120) were independent prognostic factors for LUAD (Additional file 1: Table S1).

**Fig. 2:**
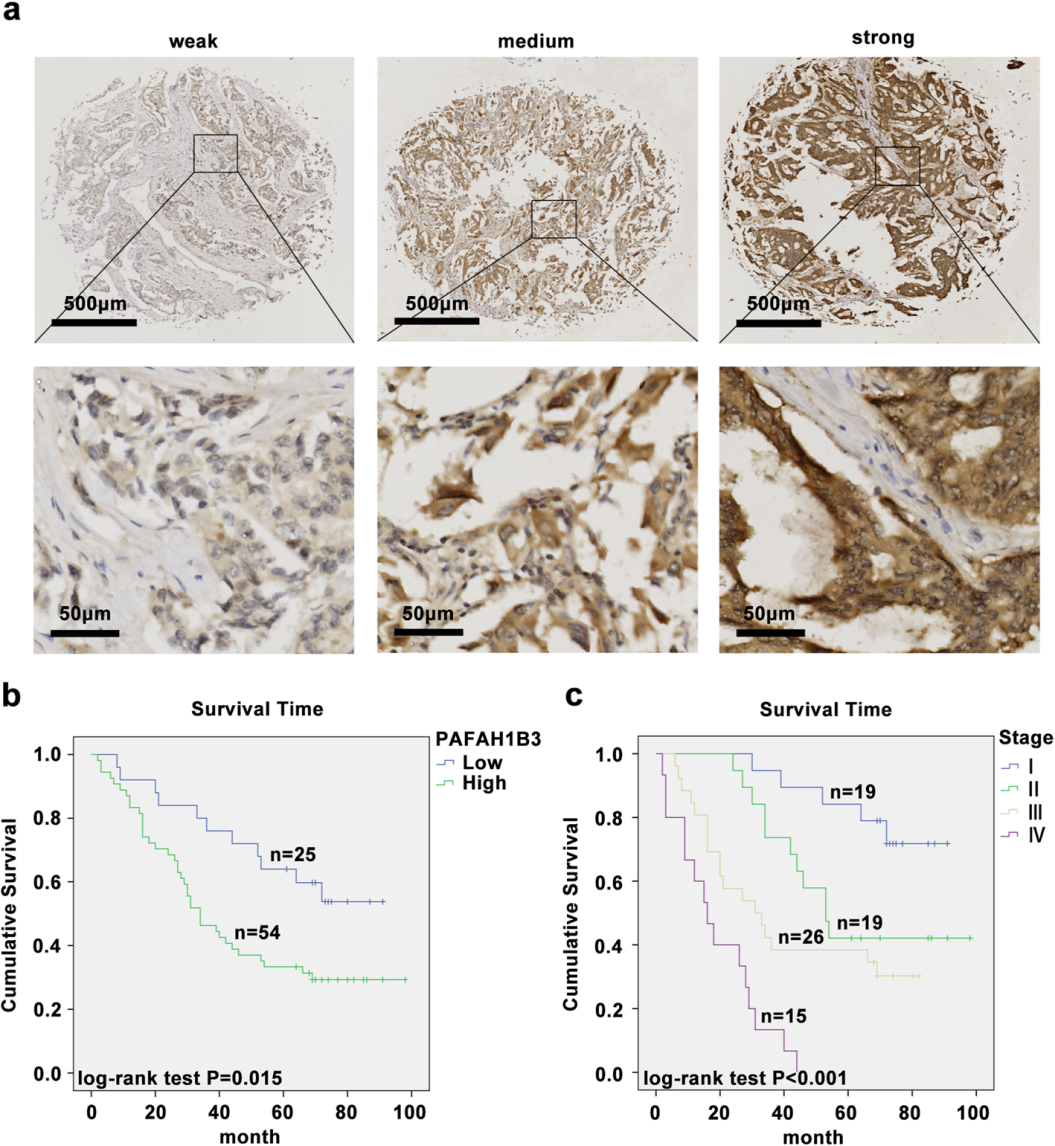
High PAFAH1B3 expression in LUAD is associated with poor survival of LUAD patients. **a** Representative immunohistochemical staining pictures for PAFAH1B3 with different staining intensity in LUAD tissues. **b** Kaplan-Meier survive curve of differential PAFAH1B3 protein expression in 79 tumor tissues in LUAD tissue microarray. **c** Kaplan-Meier survive curve of different TNM stage in 79 tumor tissues in LUAD tissue microarray

To further verify whether PAFAH1B3 expression is indeed correlated with poor clinical outcome in LUAD patients, the PAFAH1B3 protein levels in 79 LUAD patient tissue samples in a tissue microarray were quantified by IHC analyses. The staining intensity results for the LUAD tissues are shown in Fig. 2a. According to the protein expression level of PAFAH1B3, the 79 LUAD patients were divided into two categories: PAFAH1B3 low (n = 25) and PAFAH1B3 high (n = 54). We analyzed the correlation between the PAFAH1B3 expression level and clinicopathologic parameters of patients with LUAD. The χ2 test showed that the PAFAH1B3 protein expression level was remarkably positively correlated with distant metastasis (*P* = 0.021), vital status (*P* = 0.025) and TNM stage (*P* = 0.009) (Table 1). Furthermore, the univariate Cox regression analyses indicated that overexpression of PAFAH1B3 (HR = 2.240, *P* = 0.019, 95% CI = 1.141–4.394), advanced TNM stage (HR = 1937, *P* < 0.001, 95% CI = 1.431–2.621), distant metastasis (HR = 6.014, *P* < 0.001, 95% CI = 3.086–11.72) and lymph node metastasis (HR = 3.389, *P* < 0.001, 95% CI = 1.824–6.297) were prominent negative prognostic factors for patients with LUAD (Table 2). Moreover, Kaplan–Meier survival curves demonstrated that high PAFAH1B3 protein levels (log-rank *P* = 0.015) and advanced TNM stage (log-rank *P* < 0.001) were remarkably correlated with decreased OS in LUAD patients (Fig. 2b, c). Finally, multivariate Cox regression analysis further confirmed PAFAH1B3 overexpression (HR = 1.991, *P* = 0.046, 95% CI = 1.011–3.921) and advanced TNM stage (HR = 1.884, *P* < 0.001, 95% CI = 1.390–2.552) as independent prognostic risk factors for OS in LUAD patients (Table 2).

**Table 1:** Correlation between PAFAH1B3 expression and different clinicopathological Characteristics of clinical LUAD patients

Characteristics	n	PAFAH1B3		Pearson χ2	***P*** value
		Low or no expression	High expression		
**Total**	79	25(31.6)	54(68.4)		
**Age(y)**					
<60	39	13(33.3)	26(66.7)	0.101	0.75
≥ 60	40	12(30.0)	28(70.0)		
**Gender**					
Male	41	13(31.7)	28(68.3)	0.000	0.990
Female	38	12(31.6)	26(68.4)		
**Primary tumor**					
T1	20	5(25.0)	15(75.0)	0.835	0.659
T2	38	14(36.8)	24(63.2)		
T3 + T4	18	6(33.3)	12(66.7)		
Unknown	3	0(0.0)	3(100.0)		
**Lymph node metastasis**					
No	38	15(39.5)	23(60.5)	1.874	0.171
Yes	40	10(25.0)	30(75.0)		
Unknown	1	0(0.0)	1(100.0)		
**Distant metastasis**					
No	64	24(37.5)	40(62.5)	5.341	0.021*
Yes	15	1(6.7)	14(93.3)		
**TNM stage**					
stage I	19	11(57.9)	8(42.1)	11.47	0.009**
stage II	19	4(21.1)	15(78.9)		
stage III	26	9(34.6)	17(65.4)		
stage IV	15	1(6.7)	14(93.3)		
**Vital status**					
Alive	30	14(46.7)	16(53.3)	5.045	0.025*
Dead	49	11(22.4)	38(77.6)		

*P < 0.05, **P < 0.01

**Table 2:** Univariate and multivariate analysis of prognostic parameters of clinical LUAD patients using Cox regression

Characteristics	Univariate analysis			Multivariate analysis		
	HR	***P*** value	95%CI	HR	***P*** value	95%CI
**Expression of PAFAH1B3** **(low vs.high)**	2.240	0.019*	1.141–4.394	1.991	0.046*	1.011–3.921
**Age (years)** **(<60 vs. ≥ 60)**	1.394	0.248	0.793–2.450			
**Gender** **(male vs.female)**	1.320	0.335	0.751–2.320			
**Primary tumor** **(T1 vs.T2 vs.T3 + T4)**	1.104	0.632	0.736–1.658			
**Lymph node metastasis** **(No vs.Yes)**	3.389	0.000***	1.824–6.297			
**Distant metastasis** **(No vs.Yes)**	6.014	0.000***	3.086–11.72			
**TNM stage** **(I + II vs.III + IV)**	1.937	0.000***	1.431–2.621	1.884	0.000***	1.390–2.552

TNM stage contains lymph node metastasis and distant metastasis, therefore, lymph node metastasis and distant metastasis was not included in the multivariate analysis

*CI* Confidence interval, *HR* Hazard ratio. *P < 0.05, **P < 0.01, ***P < 0.001

Taken together, these results imply that high PAFAH1B3 expression correlates with advanced clinicopathological characteristics and poor prognosis in patients with LUAD and can be a prognostic factor for LUAD.

### Efficiency of lentivirus-mediated PAFAH1B3 shRNA transfection in A549 and H1299 cells

**Fig. 3:**
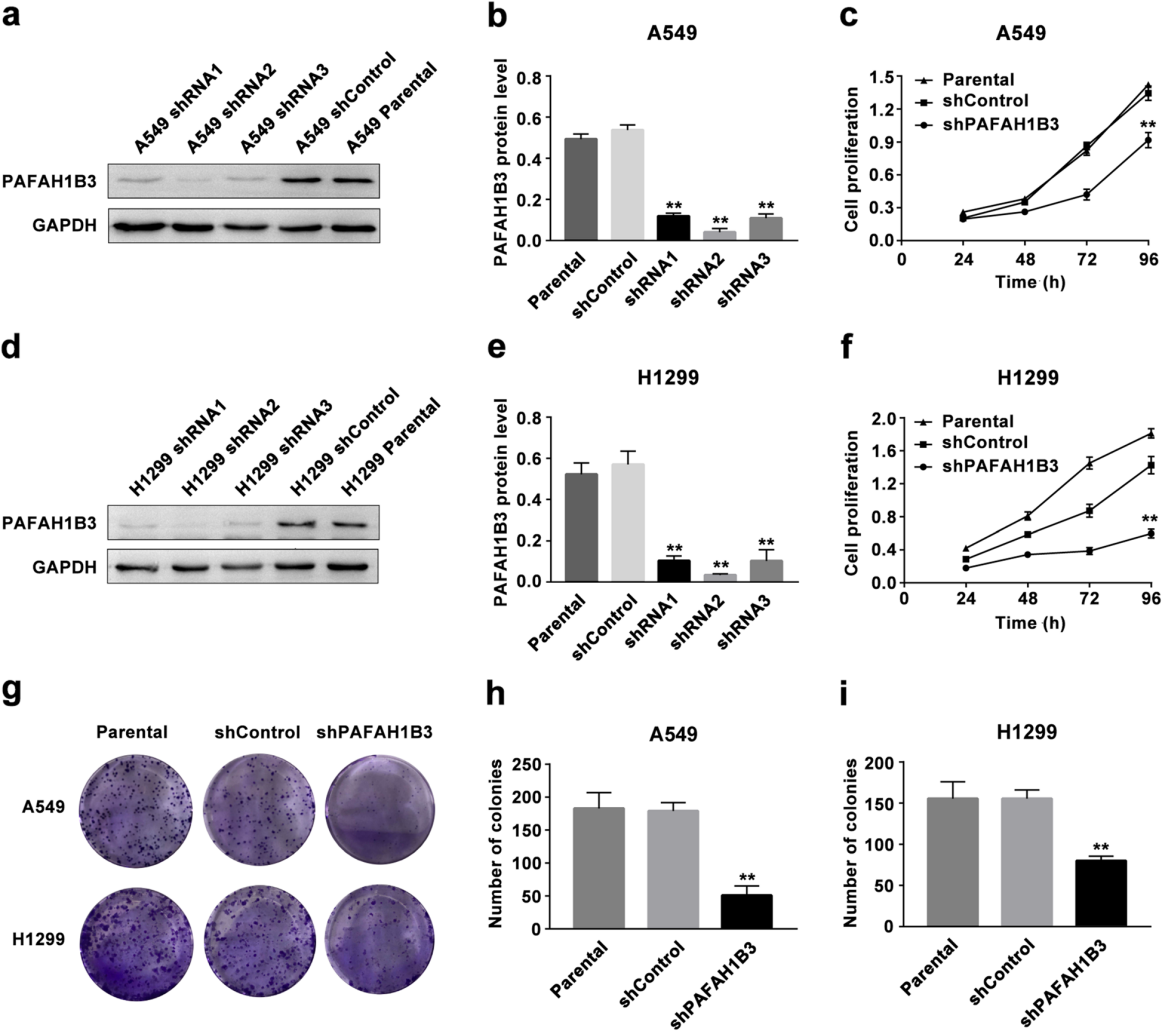
PAFAH1B3 knockdown suppresses LUAD cell proliferation *in vitro*. **a, b, d, e** PAFAH1B3 protein level in three PAFAH1B3-shRNAs of A549 and H1299 cells compared with shControl and parental cells by western blot. **c, f** CCK8 assay and (**g, h, i)** Colony formation assay were performed to measure the proliferation ability in shPAFAH1B3-transfected cells compared to shControl or parental cells. *P < 0.05, **P < 0.01

PAFAH1B3 was expressed at the highest levels in A549 and H1299 cells (Fig. 1d, e, f, *P* < 0.01); therefore, these two cell lines were selected for PAFAH1B3 shRNA transfection. Western blot analysis showed that the PAFAH1B3 protein expression level was decreased dramatically in all three shRNA groups compared with the shControl group, but shRNA2 showed the most dramatic decrease in the PAFAH1B3 protein level (Fig. 3a, b, d, e, *P* < 0.01). Therefore, A549 shRNA2 cells and H1299 shRNA2 cells were selected for subsequent experiments.

### Knockdown of PAFAH1B3 inhibits proliferation and induces G0-G1 phase arrest in A549 and H1299 cells

After stable PAFAH1B3 gene-knockdown cell lines were established, we conducted a CCK-8 assay to investigate the effect of PAFAH1B3 on LUAD cell proliferation. Silencing PAFAH1B3 dramatically reduced the growth rate of A549 and H1299 cells (Fig. 3c, f, *P* < 0.01). The results of the colony formation assay showed that shPAFAH1B3-transfected cells produced significantly smaller and fewer colonies than shControl cells (Fig. 3g, h, i, *P* < 0.01).

**Fig. 4:**
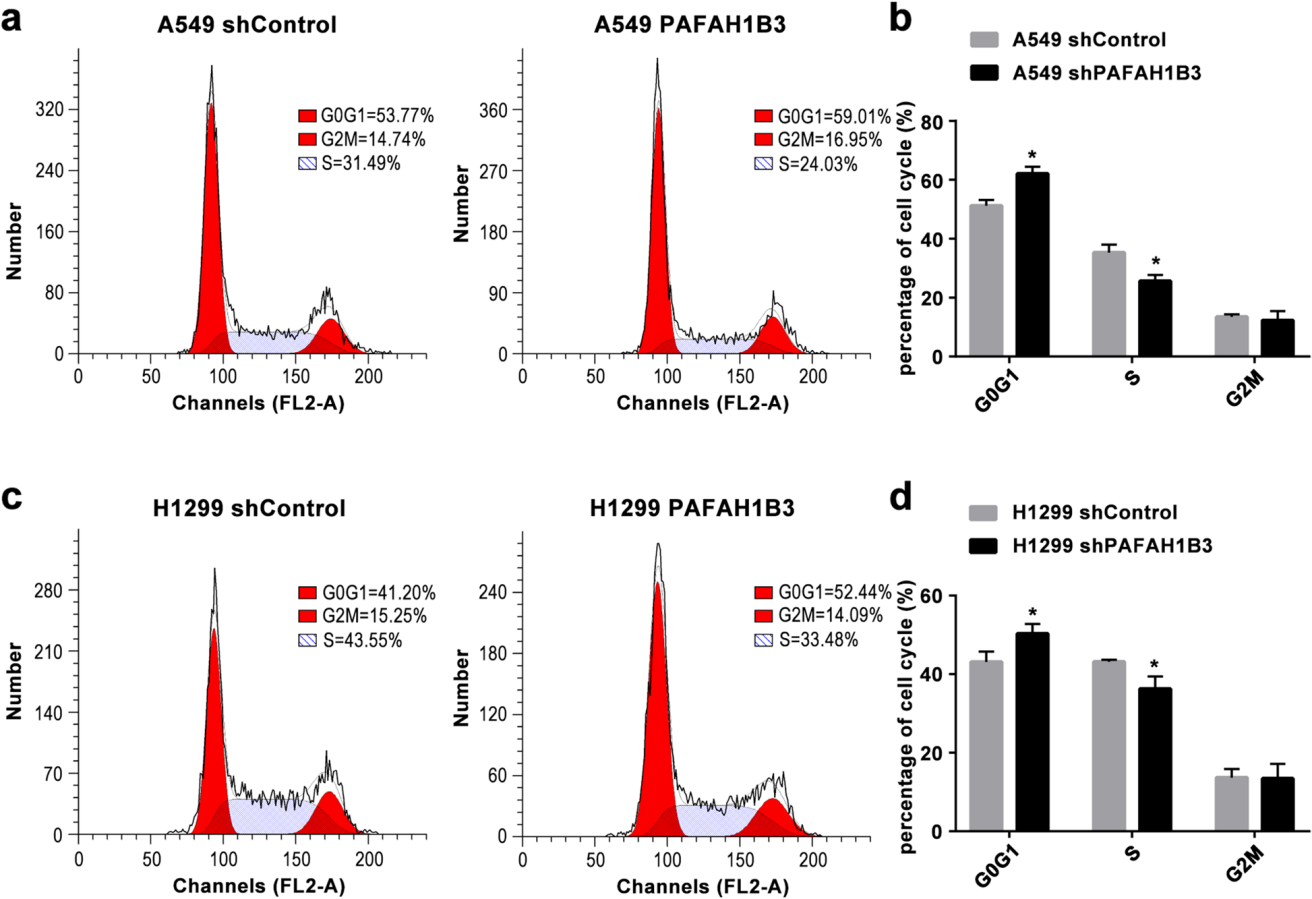
Silencing of PAFAH1B3 induces G1 phase arrest of LUAD cells. **a, c** Flow cytometry was conducted to detect the cell cycle changes in A549 and H1299 cells transfected with PAFAH1B3 shRNA2. **b, d** Statistical analysis showed increasing cell population in the G0-G1 phase and decreasing cell population in S phase. *P < 0.05

Moreover, we used flow cytometry to study the effect of PAFAH1B3 on the cell cycle of LUAD cells. The cell population in the G0-G1 phases increased while the cell population in S phase decreased in LUAD cells transfected with PAFAH1B3 shRNA (Fig. 4a, b, c, d, *P* < 0.05). These results showed that silencing PAFAH1B3 suppressed the proliferation of LUAD cells by influencing the cell cycle.

### Overexpression of PAFAH1B3 in SPCA1 cells promotes cell proliferation

As shown in Fig. 1d, SPCA1 cells manifested relatively low PAFAH1B3 expression among the cells of the four LUAD cell lines. We transfected SPCA1 cells with PAFAH1B3-overexpressing constructs to produce stable PAFAH1B3-enhanced cell lines. RT–PCR was used to verify that PAFAH1B3 was overexpressed in SPCA1-OE cells compared with control cells (Additional file 1: Fig. S3a, *P* < 0.01). We examined the proliferation of SPCA1 cells through CCK-8 assays and found that the proliferation capacity of SPCA1 cells with increased PAFAH1B3 expression was higher than that of control SPCA1 cells (Additional file 1: Fig. S3b, *P* < 0.01).

### Silencing PAFAH1B3 inhibits invasion and EMT in A549 and H1299 cells

**Fig. 5:**
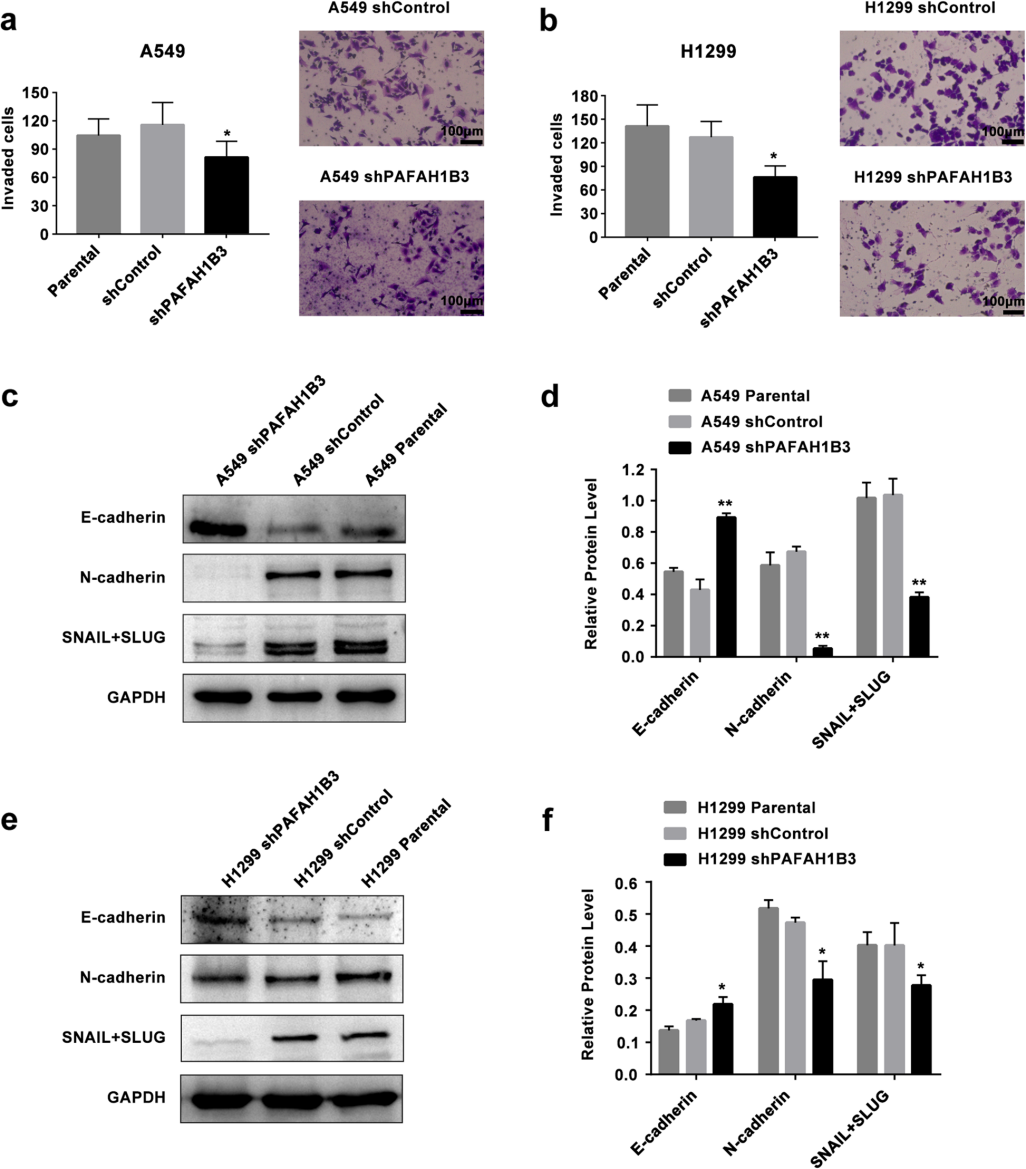
Knockdown of PAFAH1B3 inhibits invasive ability and EMT process of LUAD cells. **a, b** Invasion assay were conducted in transwell chambers pre-coated with Matrigel. **c, d, e, f** Western blot was performed to detect the protein level of EMT related markers including E-cadherin, N-cadherin and SNAIL+SLUG in PAFAH1B3 silenced cells and control cells. *P < 0.05, **P < 0.01

PAFAH1B3 expression was correlated with distant metastasis of LUAD, and one characteristic of cancer metastasis is invasion. We performed a transwell assay to assess the number of cells passing through the chambers after 48 hours of culture. The invasiveness of LUAD cells was strongly suppressed in shPAFAH1B3-transfected cells compared with shControl cells (Fig. 5a, b, *P* < 0.05).

To further explore whether PAFAH1B3 promotes LUAD cell invasion through the EMT mechanism, we measured the protein level changes in E-cadherin, N-cadherin and SNAIL+SLUG upon PAFAH1B3 downregulation. The results showed that the E-cadherin protein level was upregulated, while the N-cadherin protein level and SNAIL+SLUG protein level were downregulated in shPAFAH1B3-transfected cells compared with shControl cells and parental cells (Fig. 5c, d, e, f, *P* < 0.01). These results show that PAFAH1B3 might facilitate the invasion of LUAD cells by influencing the EMT process.

### Effect of PAFAH1B3 on tumorigenesis and EMT-related genes *in vivo*

**Fig. 6:**
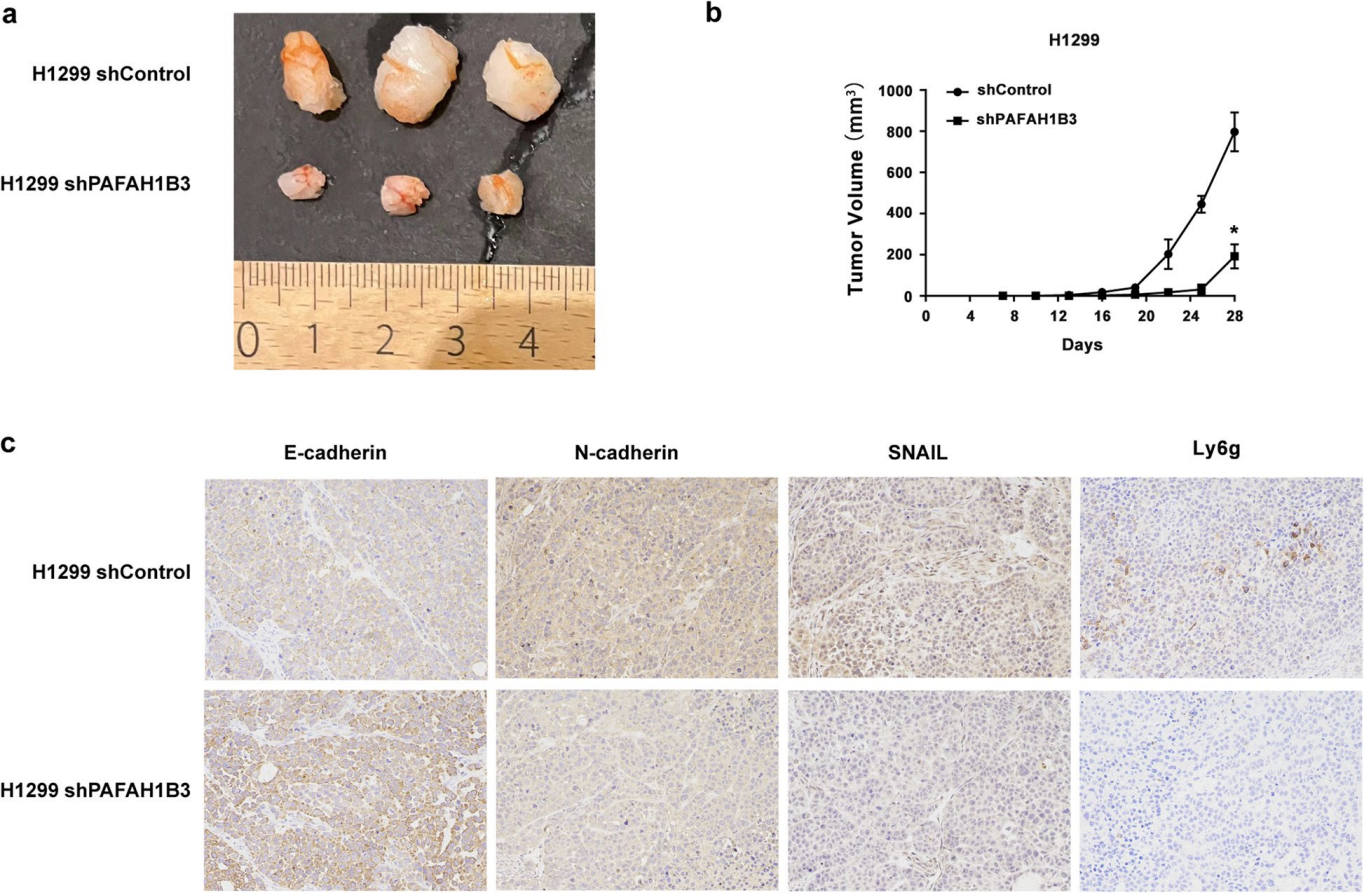
Influences of PAFAH1B3 knockdown on tumorigenesis, EMT related genes and neutrophil infiltration of H1299 cells *in vivo*. **a** Photo of xenograft tumors in shPAFAH1B3-transfected H1299 cells compared with control cells in nude mice. **b** Tumor growth curves after the mice were injected with H1299 shControl cells or PAFAH1B3 shRNA-transfected H1299 cells. **c** Expressions of E-cadherin, N-cadherin, SNAIL and Ly6g in xenograft tumors of H1299 shControl group and shPAFAH1B3 group determined by immunohistochemistry. *P < 0.05

To further evaluate the function of PAFAH1B3 *in vivo*, shControl and shPAFAH1B3 H1299 cells were injected into male nude mice to construct the xenograft model. As shown in the tumor growth curve, the growth rate of tumors was significantly slowed in the group with reduced PAFAH1B3 expression compared with the shControl group (Fig. 6b, *P* < 0.05). Furthermore, the tumor volume in the shPAFAH1B3 group was remarkably reduced compared with that in the shControl group (Fig. 6a, *P* < 0.05). Then the xenograft tumor tissues were used for IHC analyses to assess the changes in EMT-related markers after PAFAH1B3 knockdown. Consistent with the *in vitro* experiments, the expression of E-cadherin was increased, while that of N-cadherin and SNAIL was decreased in the shPAFAH1B3 group compared with the shControl group (Fig. 6c, *P* < 0.05).

### Estimation of the abundances of infiltrating immune cells

**Fig. 7:**
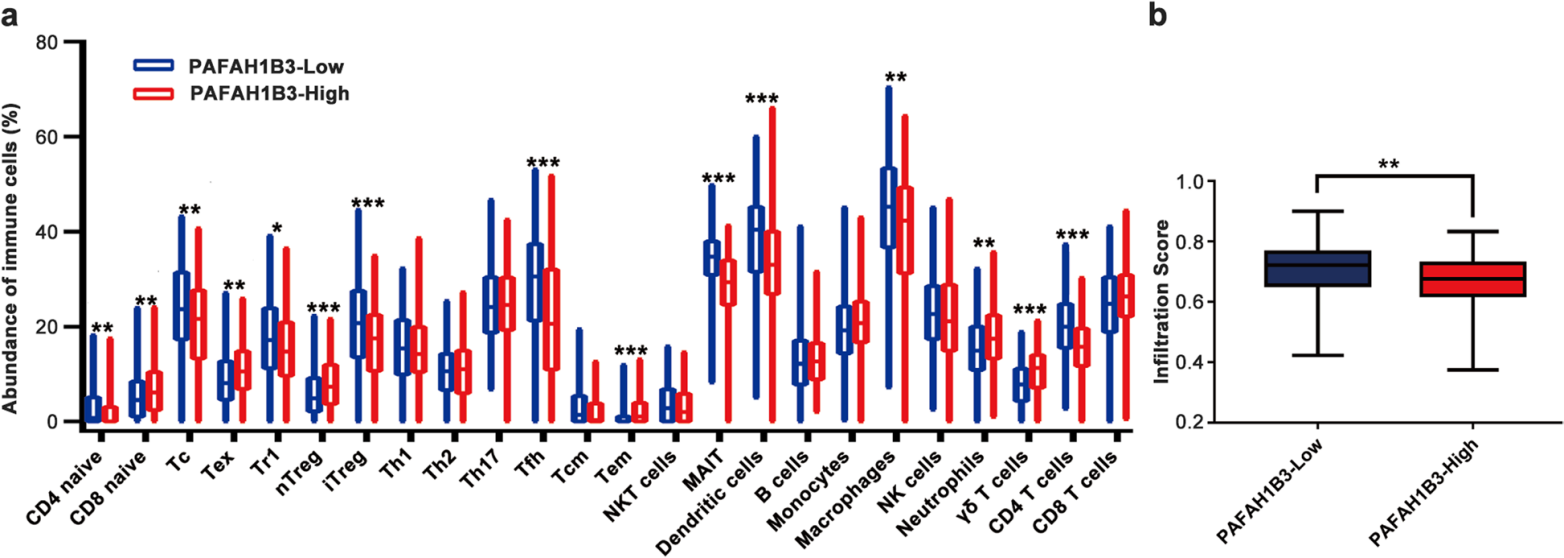
Boxplots showing the relative abundances of 24 cell types among the PAFAH1B3-high expression group and PAFAH1B3-high expression group in LUAD dataset using ImmuCellAI. **a** The relative abundances of immune cells among the PAFAH1B3-high and PAFAH1B3-Low. **b** The Immune Infiltration Score among the PAFAH1B3-high and PAFAH1B3-Low. Tc: cytotoxic T cells; Tex: exhausted T cells; Tr1: type 1 regulatory T cells; nTreg: natural regulatory T cells; iTreg: induced regulatory T cells; Th1: T helper cell type 1; Th2: T helper cell type 2; Th17: T helper cell type 17; Tfh: follicular helper T cells; Tcm: central memory T cells; Tem: effector memory T cells; NKT: natural killer T cell; MAIT: mucosal associated invariant T cells; DC: dendritic cells; NK: natural killer cells. *P < 0.05, **P < 0.01

To further explore the influence of PAFAH1B3 on the immune microenvironment of LUAD, we used ImmuCellAI to estimate the abundances of 24 infiltrating immune cells. The analysis showed that the infiltration score of the PAFAH1B3-high group was notably lower than that of the PAFAH1B3-low group (Fig. 7b, *P* < 0.01). Effector memory T (Tem) cells, CD8 naive T cells, natural regulatory T cells (nTregs), γδT cells, neutrophils and exhausted T (Tex) cells were notably enriched, while naive CD4 T cells, Tr1 cells, induced regulatory T cells (iTregs), cytotoxic T (Tc) cells, T follicular helper (Tfh) cells, mucosal-associated invariant T (MAIT) cells, dendritic cells (DCs), macrophages and CD4 T cells showed notably lower infiltration in the PAFAH1B3-high group than in the PAFAH1B3-low group (Fig. 7a). The results showed that high PAFAH1B3 expression was correlated with low levels of immune infiltration.

Among the differentially expressed infiltrating immune cells between the PAFAH1B3-high group and PAFAH1B3-low group analyzed by ImmuCellAI, neutrophils are considered the most significant predictor of poor survival in solid tumors [18]. To explore whether knockdown of PAFAH1B3 reduced neutrophil infiltration, we assessed the protein expression of neutrophil surface antigen Ly6g in xenograft tumor tissues by IHC. The suppression of PAFAH1B3 significantly reduced the expression of the neutrophil surface antigen Ly6g (Fig. 6c, *P* < 0.05).

## Discussion

PAFAH1B3, as one of the top 50 overexpressed metabolic enzymes in human cancers [19], is involved in multiple types of tumors, but the roles of PAFAH1B3 in LUAD have not been clarified. In this research, we identified PAFAH1B3 as a promising oncogene in LUAD by analyzing public datasets and clinical samples, as well as by performing functional assays *in vitro* and *in vivo*. We also found that PAFAH1B3 was correlated with immune infiltrates in LUAD.

First, we determined that PAFAH1B3 was significantly overexpressed in both LUAD tissues and cells compared with noncancerous tissues and cells. Then, we revealed that high PAFAH1B3 expression was significantly correlated with distant metastasis, advanced TNM stage and poor prognosis, indicating that PAFAH1B3 upregulation is positively related to LUAD progression. Furthermore, we confirmed that PAFAH1B3 is an independent prognostic risk factor for LUAD through multivariate Cox regression analysis. Similarly, previous studies have confirmed that there is high expression of PAFAH1B3 in HSCC tissues by IHC analyses, and high expression of PAFAH1B3 is positively correlated with poor prognosis of HSCC patients [13]. Thus, we believe that PAFAH1B3 has potential clinical value in LUAD.

Next, we conducted *in vitro* and *in vivo* experiments. We found that the suppression of PAFAH1B3 expression in LUAD cells inhibited clone formation and proliferation and caused G0-G1 phase arrest in LUAD cells and that the overexpression of PAFAH1B3 in SPCA1 cells led to enhanced proliferation of these cells. Through a xenograft tumor model, we proved that knockdown of PAFAH1B3 inhibited the tumorigenesis of LUAD cells. These results indicate that PAFAH1B3 is a novel potential oncogene in LUAD. A previous study demonstrated that knockdown of PAFAH1B3 impaired the proliferation, migration, and invasiveness of breast carcinoma cells by elevating tumor-suppressing signaling lipids [11]. Moreover, PAFAH1B3 loss *in vivo* sensitized leukemia cells to tyrosine kinase inhibitor (TKI) treatment via the platelet activating factor (PAF)/platelet activating factor receptor (PAFR) signaling pathway, and PAF/PAFR has been found to exhibit beneficial effects in some cancers [7, 12]. PAFR suppresses the inflammation and neoplastic transformation induced by chemical carcinogens [20]. PAFAH1B3 might promote malignant progression of LUAD through the PAF/PAFR signaling pathway. More studies are needed to further study the changes in downstream signaling pathways and tumor signaling lipids that occur after silencing PAFAH1B3.

Invasion is one step of metastasis. Our results demonstrated that downregulation of PAFAH1B3 significantly inhibited the invasive ability of A549 and H1299 cells. As EMT is considered the first step of metastasis [21], we further studied the mechanism through which PAFAH1B3 facilitates LUAD invasion. Through loss-of-function analysis in LUAD cells, we clearly demonstrated that PAFAH1B3 knockdown upregulated the protein expression of the epithelial marker E-cadherin but downregulated the expression of the mesenchymal marker N-cadherin and EMT-inducing transcription factors SNAIL+SLUG *in vitro* and *in vivo*.

EMT is a process by which differentiated epithelial cells transition into motile mesenchymal cells, and it involves some key events, including loss of intercellular junctions, loss of cell polarity and acquisition of enhanced cell invasion and migration capacities [22]. Tumor cells that have undergone EMT may be resistant to chemotherapy and immunotherapy, acquire stem cell properties and escape immune surveillance [23]. EMT plays a central role in the development and progression of lung cancer, and targeting EMT signaling may be a new therapeutic strategy [24].

EMT is characterized by loss of the epithelial marker E cadherin and increased expression of the mesenchymal marker N-cadherin [23]. E-cadherin expression can be repressed by EMT-related transcription factors, including SNAIL, SLUG and TWIST. SNAIL and SLUG, belonging to the snail family, are encoded by snail1 and snail2, respectively, and they can bind to the promoter region of E-cadherin and repress its transcription [25, 26]. Here, for the first time, we demonstrated that PAFAH1B3 might promote EMT by activating SNAIL+SLUG, which might lead to the downregulation of E-cadherin, resulting in enhanced invasion and mobility of LUAD cells. However, further studies of the pathways related to the activation of SNAIL+SLUG by PAFAH1B3 and studies determining whether there are other transcription factors involved in the PAFAH1B3-mediated reduction in E-cadherin are needed.

Immune cells have been increasingly recognized to be involved in carcinogenesis and cancer progression in recent years; for example, tumor-infiltrating T cells in particular have been found to significantly influence prognosis and therapeutic outcomes [27]. We demonstrated that the infiltration score was notably lower in the PAFAH1B3-high group than in the PAFAH1B3-low group, indicating that increased expression of PAFAH1B3 was correlated with a lower immune infiltration level. Furthermore, our results showed that the PAFAH1B3-high expression group had significantly enriched tumor-promoting cells, such as nTregs, exhausted T cells and neutrophils. Exhausted T cells can be observed in chronic infections and cancer, and elevated expression of inhibitory receptors on exhausted T cells facilitates CD8+ T-cell failure and immune evasion [28]. nTregs, a subpopulation of T cells, are increased in the tumor microenvironment and are associated with poor survival in lung cancer [29]. Neutrophils are considered the most significant predictor of poor survival in solid tumors [18]. We performed immunohistochemical(IHC) assays for the relevant neutrophil surface antigen Ly6g in an *in vivo* tumorigenicity model. We found that the expression level of Ly6g was significantly decreased in H1299 shPAFAH1B3 compared with H1299 shControl. Previous experiments confirmed that neutrophil infiltration promoted tumor growth, increased the ability of cancer cells to metastasize and elevated the expression of snail, which in turn accelerated neutrophil accumulation in a mouse model of LUAD [30]. These results are consistent with our results showing that neutrophils were significantly decreased while SNAIL decreased upon knockdown of PAFAH1B3. As discussed above, knockdown of PAFAH1B3 could cause reversal of EMT toward a reduced mesenchymal phenotype. Taken together, these results suggested that PAFAH1B3 affects neutrophil infiltration. Considering that neutrophil infiltration is associated with EMT, we speculate that the mechanism by which PAFAH1B3 affects the EMT process might be immune-related.

In addition, we also found that CD4+ T cells, Tc cells, DCs, Tfh cells and MAIT cells were notably enriched in the PAFAH1B3-low group, which is consistent with their positive role in antitumor processes [31–34]. Tc cells are the main cells that participate in cancer cell killing and tumor elimination [31]. With the help of special DCs, CD4+ T cells can relay helpful signals to enhance the antitumor properties of cytotoxic T lymphocytes [32]. These results may partly account for the poor clinical outcomes of LUAD patients with PAFAH1B3 overexpression, and more experiments are needed to confirm the association between PAFAH1B3 expression and tumor-infiltrating immune cells.

We concluded that PAFAH1B3 promoted proliferation, invasion and EMT and affected immune infiltrates in LUAD. And we also consider exploring whether inhibition of EMT could inhibit proliferation, invasion and migration in the future. Our experiments indicated that PAFAH1B3 plays an oncogenic role in the progression of LUAD and might be a reasonable target for LUAD therapy. In the future, specific biological agents targeting PAFAH1B3 could be developed to benefit LUAD patients with high PAFAH1B3 expression.

## Conclusion

In summary, we not only confirmed the oncogenic role of PAFAH1B3 in LUAD but also partially explained that PAFAH1B3 participates in LUAD progression through regulating EMT signaling pathway; we also found the relationship between PAFAH1B3 expression and infiltrating immune cells. Our research establishes a solid theoretical foundation for further in-depth research regarding the mechanism by which PAFAH1B3 facilitates LUAD malignancy.

## Supplementary Information

**Additional file 1: Figure S1.** PAFAH1B3 expression is up-regulated in LUAD tissues. **Figure S2.** High PAFAH1B3 expression in LUAD is associated with poor survival of LUAD patients. **Figure S3.** Overexpression of PAFAH1B3 in SPCA1 cells promote cell proliferation in vitro. **Table S1.** Univariate and multivariate analysis of prognostic parameters in TCGA LUAD patients using Cox regression.

## Data Availability

The datasets analysed during the current study are available in the Cancer Genome Atlas (TCGA, http://cancergenome.nih.gov/) and the Gene Expression Profiling Interactive Analysis (GEPIA, http://gepia.cancer-pku.cn/).

## Abbreviations

PAFAH1B3: Platelet-activating factor acetylhydrolase 1b catalytic subunit 3
LUAD: Lung adenocarcinoma
GEPIA: Gene Expression Profiling Interactive Analysis
RT-PCR: Real-time PCR
IHC: Immunohistochemical
TCGA: the Cancer Genome Atlas Program
ImmuneCellAI: Immune Cell Abundance Identifier
EMT: Epithelial-to-mesenchymal transition
NSCLC: Non-small-cell lung cancer
OS: Overall survival
DFS: Disease free survival
PAF-AHs: platelet-activating factor acetylhydrolases
PAF: Platelet-activating factor
HSCC: Hypopharyngeal squamous cell carcinoma
Tc: Cytotoxic T cells
Tex: Exhausted T cells
Tr1: Type 1 regulatory T cells
nTreg: Natural regulatory T cells
iTreg: Induced regulatory T cells
Th1: T helper cell type 1
Th2: T helper cell type 2
Th17: T helper cell type 17
Tfh: Follicular helper T cells
Tcm: Central memory T cells
Tem: Effector memory T cells
NKT: Natural killer T cell
MAIT: Mucosal associated invariant T cells
DC: Dendritic cells
NK: Natural killer cells
TKI: Tyrosine kinase inhibitor
PAFR: Platelet activating factor receptor
