# Supplementary material for: PAFAH1B3 predicts poor prognosis and promotes progression in lung adenocarcinoma
Source: BMC Cancer. 2022 May 9;22:525. doi: 10.1186/s12885-022-09617-x (PMC9087959; doi:10.1186/s12885-022-09617-x)
Supplement: Supplementary file 1 — Additional file 1: Figure S1. PAFAH1B3 expression is up-regulated in LUAD tissues. Figure S2. High PAFAH1B3 expression in LUAD is associated with poor survival of LUAD patients. Figure S3. Overexpression of PAFAH1B3 in SPCA1 cells promote cell proliferation in vitro. Table S1. Univariate and multivariate analysis of prognostic parameters in TCGA LUAD patients using Cox regression. [file 12885_2022_9617_MOESM1_ESM.pdf]

Supplementary material

Title page

PAFAH1B3 predicts poor prognosis and promotes progression in lung adenocarcinoma

Suping Tang[1,5], Jun Ni[2, 3], Bohua Chen[4], Fei Sun[1], Jinbo Huang[1], Songshi Ni[1,], Zhiyuan Tang[4,*]*

[1] *Department of Respiratory and Critical Care Medicine, Affiliated Hospital of Nantong University, Nantong 226001, Jiangsu, China*

[2] *Department of Rehabilitation Medicine, The First Affiliated Hospital of Fujian Medical University, Fuzhou 350000 Fujian, China*

[3] *Department of Rehabilitation Medicine, Affiliated Hospital of Nantong University, Nantong 226001, Jiangsu, China*

[4] *Department of Pharmacy, Affiliated Hospital of Nantong University, Nantong 226001, Jiangsu, China*

[5] *Department of Respiratory and Critical Care Medicine, The Affiliated Wuxi Second People's Hospital of Nanjing Medical University, Wuxi 214000, Jiangsu, China*

Correspondence: *Songshi Ni, Tel: +86-13706293196, Fax: +86-513- 85519820, E-mail: jsntnss@163.com or Zhiyuan Tang, Tel: +86-18862804577, Fax: +86-513-85052230, E-mail: tina2951@sina.com*

Supplementary Figure

Fig. S1 *PAFAH1B3 expression is up-regulated in LUAD tissues. (a) PAFAH1B3 mRNA expression in the LUAD tissues (483 samples) compared with the normal lung tissues (347 samples) by GEPIA. (b) PAFAH1B3 mRNA expression in 57 paired lung tumor samples in TCGA-LUAD datasets.*

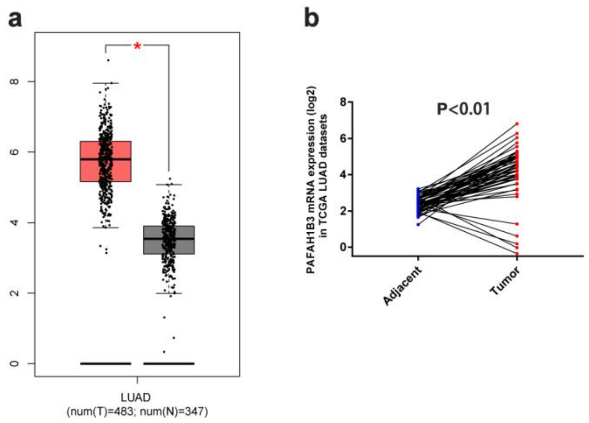

Fig. S2 *High PAFAH1B3 expression in LUAD is associated with poor survival of LUAD patients. (a) Overall Survival curve of differential PAFAH1B3 mRNA expression by GEPIA. (b) Disease Free Survival curve of differential PAFAH1B3 mRNA expression by GEPIA.*

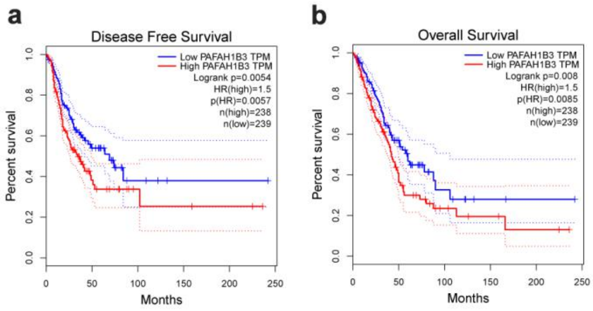

Fig. S3 Overexpression of PAFAH1B3 in SPCA1 cells promote cell proliferation in vitro. (a) PAFAH1B3 mRNA level in PAFAH1B3-OE SPCA1 cells compared with Control SPCA1 cells by RT-PCR. (b) CCK8 assay was performed to measure the proliferation ability in PAFAH1B3-OE SPCA1 cells compared to Control SPCA1 cells. *P < 0.05, **P < 0.01

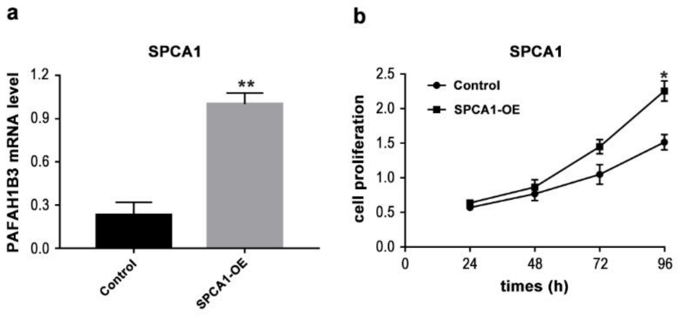

Supplementary Table

Table S1. *Univariate and multivariate analysis of prognostic parameters in TCGA LUAD patients using Cox regression.*

Characteristics	Univariate analysis			Multivariate analysis		
	HR	P value	95%CI	HR	P value	95%CI
**Expression of PAFAH1B3 (low vs.high)**	*1.497*	*0.009***	*1.108-2.023*	*1.416*	*0.024**	*1.047-1.915*
**Age (years) (<60 vs.≥60)**	*1.078*	*0.662*	*0.796-1.511*			
**Gender (male vs.female)**	*0.900*	*0.486*	*0.668-1.212*			
**Primary tumor (T1 vs.T2 vs.T3+T4)**	*1.690*	*0.000****	*1.336-2.139*			
**Lymph node metastasis (No vs.Yes)**	*2.496*	*0.000****	*1.851-3.366*			
**TNM stage (Ⅰ vs.Ⅱ vs.Ⅲ+Ⅳ)**	*1.804*	*0.000****	*1.517-2.145*	*1.782*	*0.000****	*1.498-2.120*

TNM stage contains lymph node metastasis and primary tumor, therefore, lymph node metastasis and primary tumor was not included in the multivariate analysis.

*CI: Confidence interval; HR: Hazard ratio. *P<0.05, **P<0.01, ***P<0.001*
